# Heart Failure and Hypothermia in an Infant: Pseudocyanide Syndrome?

**DOI:** 10.1155/2018/2895124

**Published:** 2018-04-01

**Authors:** J. Scott Baird

**Affiliations:** Department of Pediatrics, Division of Critical Care Medicine, Columbia University, New York, NY, USA

## Abstract

**Purpose:**

Mixed or central venous oxygen saturation has not been described during concurrent heart failure and hypothermia in children, both of which may be associated with hyperlactatemia. This report of an infant with heart failure and hypothermia is significant for increased inferior vena cava (IVC) oxygen saturation and hyperlactatemia.

**Case Report:**

A 36-day-old female was fussy for a day and then developed respiratory distress. In the Pediatric ER, she was tachycardic (260 beats/minute) and hypothermic (32.4 degrees C) with prolonged capillary refill and faint distal pulses. Adenosine was given twice via an intraosseous line for supraventricular tachycardia, with conversion to sinus rhythm. Blood drawn from an IVC catheter was significant for uncorrected (for temperature) oxygen saturation of 94% and lactate 18 mmol/L; corrected and uncorrected IVC oxygen saturation early during rewarming were >90%. During rewarming, declines in uncorrected IVC oxygen saturation and lactate correlated. Hypothermia and hyperlactatemia resolved after 10 and 12 hours.

**Conclusions:**

Concurrent heart failure and hypothermia in an infant were associated with increased IVC oxygen saturation and hyperlactatemia, similar to lab findings associated with a mitochondrial toxin such as cyanide. Improvement of heart failure and hypothermia were associated with resolution of these lab abnormalities, thus helping to rule out mitochondrial toxins. Additional reports may help better define a pseudocyanide syndrome in this setting.

## 1. Introduction

Mixed or central venous oxygen saturation (SO_2_) is decreased with heart failure and increased with hypothermia; though normal limits for mixed or central venous SO_2_ require interpretation within a clinical context, levels below 70% and above 90% are concerns [1]. Hyperlactatemia is common to both heart failure and hypothermia. With concurrent heart failure and hypothermia in children, hyperlactatemia is increased in proportion to heart failure severity [2], but mixed or central venous SO_2_ data are lacking. This report of an infant with heart failure and hypothermia is notable for increased inferior vena cava (IVC) SO_2_ and hyperlactatemia, similar to the findings associated with a mitochondrial toxin, such as cyanide.

## 2. Case Report

The Columbia University Medical Center IRB (AAAR0802) exempted this retrospective case report from review.

**Figure 1:**
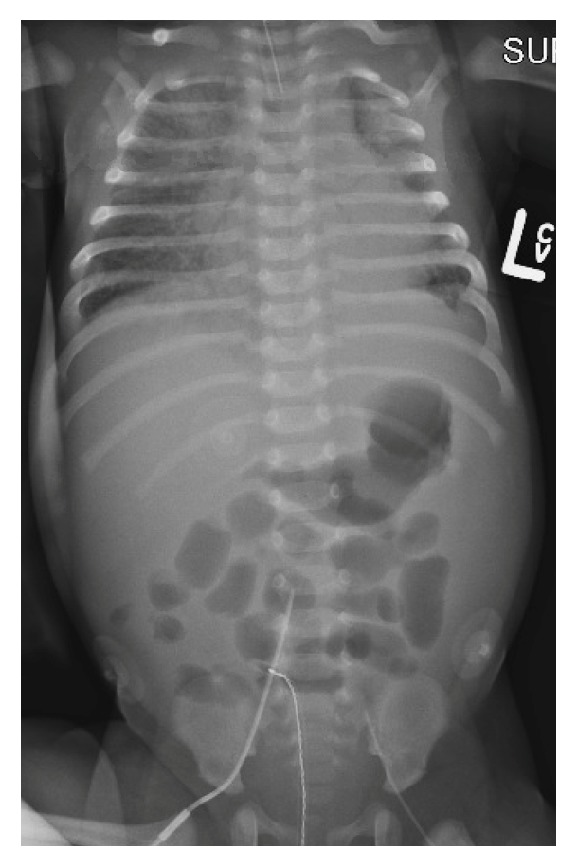
Radiograph of infant with heart failure and mild hypothermia showing central venous catheter overlying L3 vertebral body.

**Figure 2:**
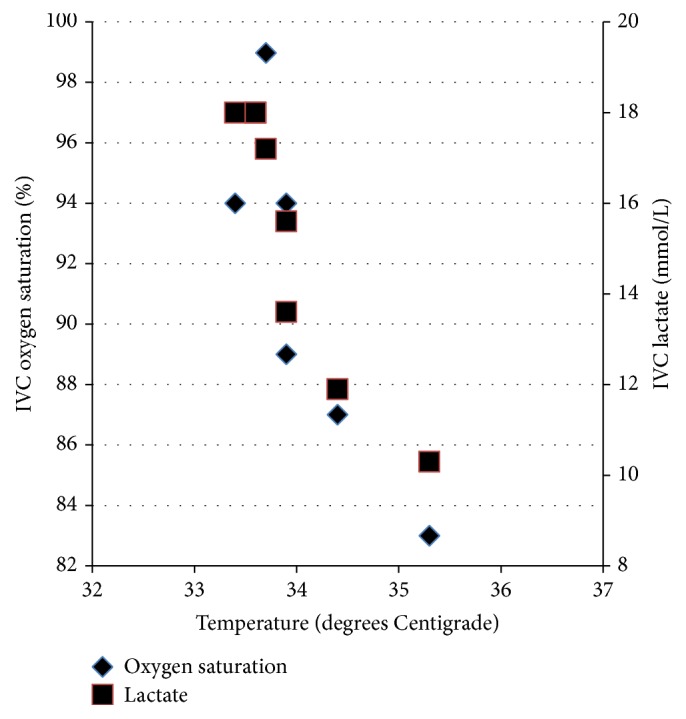
Inferior vena cava oxygen saturation (uncorrected for temperature) and lactate during rewarming of infant with heart failure and mild hypothermia.

A 36-day-old female was fussy for a day and then developed respiratory distress. She was born at term, and a New York State newborn screen was negative. In the Pediatric ER, she was tachycardic (260 beats/minute) and hypothermic (32.4 degrees C) with prolonged capillary refill and faint distal pulses. She was placed on continuous positive airway pressure via nasal prongs. Adenosine was given twice via an intraosseous line for supraventricular tachycardia with conversion to sinus rhythm. She was then intubated for worsening respiratory distress and placed on an FiO_2_ of 1.0. An echocardiogram was notable for decreased biventricular systolic function and left to right shunting through a patent foramen ovale. A dobutamine infusion was started. Arterial cannulation was unsuccessful; transcutaneous pulse SO_2_ was consistently 100% during rewarming. Results from the first blood gas (all blood gas results were reported uncorrected for temperature) drawn from a catheter placed in the IVC (by Seldinger technique; sutured in place after advancing to its full length overlying the L3 vertebral body; Figure 1) were pH 7.01, PCO_2_ 33 mm Hg, PO_2_ 101 mm Hg, SO_2_ 94%, and lactate 18 mmol/L. Early during rewarming, the IVC SO_2_ remained above 90% and was as high as 99% (Figure 2); declines in IVC SO_2_ and lactate during the first 3 hours of rewarming correlated (Spearman *r*: 0.94; *p* = 0.002). An arterial catheter was then placed, and the FiO_2_ weaned. B-type natriuretic peptide was increased (4578.5 pg/mL). Capillary refill and distal pulses improved, and dobutamine was discontinued at 8 hours; a repeat echocardiogram the next day showed improved (though still diminished) biventricular systolic function with continued left to right shunting through a patent foramen ovale. Hypothermia and hyperlactatemia resolved over 10 and 12 hours, and IVC SO_2_ was 80% on hospital day 2. The patient was diagnosed with supraventricular tachycardia secondary to Wolff-Parkinson-White syndrome. Prior to discharge a week later, serum acylcarnitine profile, lactate, pyruvate, thyroid stimulating hormone, and thyroxine were all normal.

IVC PO_2_ values were corrected retrospectively for the patient's temperature [3] (Table 1).

## 3. Discussion

Concurrent acute heart failure and mild (>32 degrees Centigrade [4]) hypothermia were associated with an increased SO_2_, profound metabolic acidosis, and severe hyperlactatemia in blood from the IVC of an infant. Heart failure alone is associated with a decreased mixed venous or central venous SO_2_—as well as a decreased IVC SO_2_—in neonates [5] as well as infants and children; mild hypothermia alone is associated with an increased—though still normal—mixed venous [6] or central venous [7] SO_2_ and is unlikely to be responsible for profound acidosis [4] or severe hyperlactatemia [8, 9]. Resolution of heart failure and mild hypothermia in this infant was associated with resolution of the lab abnormalities: a decrease in cardiac output and tissue metabolic rate, as well as peripheral vasoconstriction and vascular shunting, all improved with resolution of heart failure and hypothermia. Similar lab abnormalities are associated with cyanide toxicity [10, 11], though the pathophysiology is different: as oxidative phosphorylation is disrupted, oxygen extraction and consumption decrease due to the metabolic block, and venous SO_2_ increases with hyperlactatemia. Though a metabolic disease or toxin might have been associated with the clinical presentation of this infant, there was ultimately no evidence to support such a diagnosis or its associated pathophysiology.

A clinical controversy regarding blood gas results may have escaped the notice of some pediatric intensivists: correcting blood gas results for the patient's temperature is no longer routine, as recommended [12]. As a result, blood gas specimens are routinely analyzed at 37 degrees Centigrade (normothermia) and so reported. Interestingly, correcting the blood gas results retrospectively in this infant did not alter the findings: early during rewarming (on the first 3 blood gas results), the corrected IVC PO_2_ was >80 mm Hg, corresponding generally to SO_2_ values on the flat upper part of the oxyhemoglobin dissociation curve. As calculations of SO_2_ from PO_2_ may be associated with inaccuracies [13], specific SO_2_ values were not calculated; however, a leftward shift of the oxyhemoglobin dissociation curve (due both to hypothermia and the presence of fetal hemoglobin in a 5 week old infant) supports IVC SO_2_ values > 90% for PO_2_ values > 80 mm Hg. It is thus evident that both corrected and uncorrected IVC SO_2_ values early during rewarming were increased above normal.

The IVC SO_2_ was >90% in 27% (15 of 55) of values from 20 critically ill neonates and infants undergoing cardiopulmonary bypass (mostly with hypothermia), though lactate was only mildly elevated (mean: 2.2 +/− 0.1 mmol/L) [14]. As cardiopulmonary bypass replaces cardiac function, it is possible that heart failure with mild hypothermia in an infant would be associated with more severe hyperlactatemia and an increased IVC SO_2_, as noted in this infant. In any case, the trend in lab results from the IVC catheter was consistent during rewarming following cardioversion and suggests that these findings represent an actual association.

Limitations to this report include those related to retrospective case reports generally. Some laboratory data—including arterial blood gas results as well other metabolic testing—were not available due to limited vascular access during acute illness. The FiO_2_ was maintained at 1.0 during the first 3 hours of rewarming while the infant's clinical condition stabilized, and this could have contributed to an increased IVC SO_2_; however, this effect was not dramatic, as the IVC SO_2_ fell to 83% before the FiO_2_ was weaned. In any case, pediatric intensivists may benefit from knowing that the SO_2_ from a femoral vascular catheter may be as high as 99% in certain clinical conditions, even when the catheter is not arterial. As the IVC catheter was advanced to its full length and sutured in place overlying the L3 vertebral body, proximal catheter migration was not possible, such that the left to right shunt across the patent foramen ovale did not contribute to an increased IVC SO_2_.

## 4. Conclusion

This report offers anecdotal evidence that IVC SO_2_ may not always be low in infants with heart failure, while lactic acidosis may be profound in the presence of mild hypothermia: concurrent heart failure and mild hypothermia were likely responsible for the lab abnormalities in this infant, which to some extent mimic the lab abnormalities associated with a mitochondrial toxin such as cyanide. Further study of a pseudocyanide syndrome associated with concurrent heart failure and mild hypothermia in infants may be helpful.

## Figures and Tables

**Table 1:** Temperature, uncorrected and corrected (for temperature) IVC PO_2_, uncorrected IVC SO_2_, and IVC lactate in infant with heart failure and mild hypothermia.

Time (minutes)	Temperature (degrees Centigrade)	Uncorrected PO_2_ (mm Hg)	Corrected PO_2_ (mm Hg)	Uncorrected SO_2_ (%)	Lactate (mmol/L)
0	33.4^*∗*^	101	83	94	18
63	33.6^*∗*^	100	84	97	18
74	33.7^*∗*^	174	134	99	17.2
87	33.9^*∗*^	71	58	94	15.6
106	33.9	59	48	89	13.6
133	34.4	60	50	87	11.9
177	35.3^*∗*^	49	44	83	10.3

^*∗*^Interpolated temperatures.
